# Unraveling the Mesoscale Evolution of Microstructure during Supersonic Impact of Aluminum Powder Particles

**DOI:** 10.1038/s41598-018-28437-3

**Published:** 2018-07-04

**Authors:** Sumit Suresh, Seok-Woo Lee, Mark Aindow, Harold D. Brody, Victor K. Champagne, Avinash M. Dongare

**Affiliations:** 1 Department of Materials Science and Engineering, Institute of Materials Science, University of Connecticut, 97 North Eagleville Road, Storrs, CT 06269 USA; 2 U.S. Army Research Laboratory, Weapons and Materials Research Directorate, Aberdeen Proving Ground, Aberdeen, MD 21005 USA

## Abstract

A critical challenge in the predictive capability of materials deformation behavior under extreme environments is the availability of computational methods to model the microstructural evolution at the mesoscale. The capability of the recently-developed quasi-coarse-grained dynamics (QCGD) method to model mesoscale behavior is demonstrated for the phenomenon of supersonic impact of 20 µm sized Al particles on to an Al substrate at various impact velocities and over time and length scales relevant to cold spray deposition. The QCGD simulations are able to model the kinetics related to heat generation and dissipation, and the pressure evolution and propagation, during single particle impact over the time and length scales that are important experimentally. These simulations are able to unravel the roles of particle and substrate deformation behavior that lead to an outward/upward flow of both the particle and the substrate, which is a likely precursor for the experimentally observed jetting and bonding of the particles during cold spray impact.

## Introduction

The lack of available computational methodologies to model microstructural evolution at the mesoscales under extreme environments is a critical bottleneck in our ability to design materials and processes for improved performance^1^. While most of the advances in scientific insights using modeling methodologies have focused on materials behavior and phenomena at the nanoscale, the fundamental understanding of the collective distribution and evolution of defects and interfaces at the mesoscales is still unclear. Of particular importance is the macroscale behavior of materials under extreme environments of shock, impact, high strain rates, etc. that relies not only on the atomic level nucleation and evolution of defects (dislocations, faults, etc.) and their interaction with grain boundaries and interfaces, but also on the collective interaction and evolution of these defects to determine the macroscale deformation response. The ability to model such a collective description of microstructural evolution in these environments, while retaining the atomic level physics of the processes involved, poses both an unprecedented challenge and a unique opportunity to design and manufacture materials at the mesoscales. The validation of such capabilities, in addition, requires experimental characterization of the phenomena and microstructures at the same length and time scales.

Such an opportunity to model deformation behavior and characterize post-impact microstructures at the same length and time scales is provided by the cold spray (CS) process. This process involves accelerating powder particles to supersonic velocities before they impinge upon a target substrate, causing bonding of the particles to the substrate^2^. The metal powder particles are typically a few tens of microns in diameter, and the phenomenon of single particle impact spans time scales of up to a hundred nanoseconds during CS^3^. The CS process has recently emerged as a viable means to form coatings, to provide dimensional restoration and/or to produce near net shape parts from various metallic, polymer and ceramic systems^4,5^. A critical challenge faced in the optimization of this process is the lack of a fundamental understanding of the microstructural evolution in the particle and at the substrate surface during the supersonic impact^6^.

The current experimental understanding of the process of single particle impact suggests several mechanisms including mechanical interlocking^7,8^, rupture of the surface oxide layers of the particle and substrate^9,10^, adiabatic shear instability^11–13^, and localized melting^14–18^ to result in metallurgical bonding at the particle/substrate interface. Although the bonding mechanisms involved may vary depending on the deformability of the substrate and the particle and process parameters, it is now well established that adhesion at the substrate/particle interface occurs only beyond a “critical impact velocity”^19–28^, which depends on the particle size, temperature, microstructure and other material properties like melting temperature^20,22,24,26,29–31^ and is also associated with “jetting”, i.e. material ejection at the substrate/particle interface. Several modelling approaches have been explored to gain insights into the deformation behavior of the particle and substrate during cold spray impact and into the mechanisms related to bonding of the particle. Since the dimensions of the powder particles are on the order of tens of microns, most of these studies are based on finite element modeling (FEM)^11–13,19–24,32–39^. In most of these models, this critical velocity is computed as being the velocity at which the onset of the “adiabatic shear instability” (ASI) occurs at the substrate/particle interface. Since high velocity impacts during CS lead to material deformation at high strain rates, competing events like strain hardening and thermal softening due to heat generation are expected to occur. This latter softening effect plays a critical role in the regions near the substrate/particle interface leading to localization of high temperature regions and consequently giving rise to a shear instability and material flow in this region as the softening rates exceed the hardening rates^12^. It has been established that the onset of ASI is a size-dependent phenomenon^22^ with the larger particles showing ASI more readily. Here, a measure of the percentage of the contact area that has undergone ASI shows the extent of the bonding that has taken place. While the definition of bonding has been an inherent problem in many of these models, ASI has now become a more commonly accepted phenomenon that can define the onset of bonding in CS and can thereby be used to determine the critical velocity for a particular system. However, a critical challenge in the applicability of these methods is the inherent limitations of the flow stress models^40–44^. These limitations arise because many of the material parameters and behaviors (defect degeneration, strain rate dependence, temperature evolution, melting, etc.) required for these models are not available under loading conditions that prevail during CS impact (strain rates of up to 10^9^ s^−1^)^5,20^. As a result, the choice of flow stress model leads to variations in the deformation behavior of the particles and hence the capability to predict “jetting” behavior^45^. Discrepancies have also been observed for variations in mesh-size dependence^46^ and in user defined inputs to control whether the particle should rebound or adhere to the substrate following impact^20^. Discrete element modeling methods like smoothed-particle hydrodynamics (SPH)^47–49^ have also been used to investigate the bonding mechanisms during single particle impact. While the use of SPH models avoids mesh-related inconsistencies, such models may lack the spatial resolution required to capture the interfacial phenomena that determine the softening and deformation behavior of the particle and the substrate during impact^46^.

Thus, investigations of the bonding mechanisms require accurate representations of the microstructural evolution during deformation of the powder particles and the substrate, and of the related evolution of temperature and pressures at the particle/substrate interface during single particle impact. Classical molecular dynamics (MD) simulations have the capability to investigate the microstructural response of powder particles and the substrate during impact at the atomic scales. For example, MD simulations can provide critical insights into the nucleation and evolution of defects (dislocations, stacking faults, etc.), and into the temperatures and pressures at the particle/substrate interface^50–57^. However, the applicability of MD simulations to understand the deformation behavior, bonding characteristics and ASI is limited to significantly smaller time and length scales than those which prevail in the experiments. The current state-of-the-art simulations enable modeling of systems with length scales of a few hundred nanometers and time scales of a few nanoseconds^58^ as compared to powder particles that span tens of microns in diameter and the phenomenon of a single particle impact that spans time scales of up to a hundred nanoseconds during CS^5^. Since it has now been established that the bonding characteristics of the particle during CS impact are correlated to the sizes of the particles, with the larger particles showing ASI^22^, it is relevant to note that there has thus far been no evidence of ASI reported using MD simulations^5^.

Thus, opportunities exist for the development of capabilities that can bridge the mesoscale gap between the MD and continuum simulations, thereby unravelling the atomic scale physics of the deformation processes that determine the evolution of microstructure and the formation of a metallurgical bond at the experimental scales. The “quasi-coarse-grained dynamics” (QCGD)^59^ method is one such method that is able to bridge this gap by coarse-graining the atomic scale microstructure and using representative atoms (R-atoms) to mimic the dynamics of several atoms in a given volume of an atomistic microstructure. Such a framework requires scaling relationships for the atomic scale energetics of the R-atoms (and degrees of freedom to account for the missing atoms in the microstructure) based on the embedded atom method (EAM) interatomic potential for Al^60^ used in MD simulations. These scaling relationships are defined to represent a chosen volume of atomic scale unit cells by coarse-grained (CG) unit cells such that the length scales of the microstructural features and the time scales of the phenomena can be modeled in the CG microstructure. Such scaling relationships are able to incorporate MD-predicted kinetics related to collective nucleation, evolution and interaction mechanisms of dislocations, generation of pressures and temperatures during the course of a simulation, as well as phase transformation behavior in metallic materials^61–63^. The QCGD simulations are able to model the shock deformation and spall failure of polycrystalline microstructures of metals at the length scales of tens of microns and provide critical insights in defect nucleation and evolution behavior during these processes^62^. Hence, the scope of the QCGD technique allows “atomistic properties” (temperature, pressure, atomic stresses and velocities, dislocation densities *etc*.) and “atomistic mechanisms” to be tracked as compared to the information that can be obtained from a continuum based model like FEM. The capability of the QCGD simulations to model the MD-predicted dynamic evolution of microstructure during single particle impact using a significantly reduced number of R-atoms and scaling relationships for the atomic scale interatomic potentials and the degrees of freedom is demonstrated in Supplementary Figs 1 and 2. More details about the QCGD scaling relationships used here are provided in the “Materials and Methods” section and in the Supplementary Notes 1 and 2.

The QCGD simulations discussed here are used to investigate the dynamic evolution of pressure and temperature at the particle/substrate interface during single particle impact of a 20 µm polycrystalline Al powder particle onto a polycrystalline Al substrate at various impact velocities ranging from 700 m/s to 1600 m/s. More details of the scaling relationships used at these length scales are provided in Supplementary Note 3, and the ability to retain the mechanical behavior and thermodynamic behavior is demonstrated in Supplementary Fig. S3. The simulation setup to model cold spray impact of individual Al particles is discussed in Supplementary Note 4 and is shown in Supplementary Fig. S4. As a first approximation, the Al particle (yellow) is equilibrated at a temperature of ~530 K corresponding to a heated gas stream, and the substrate is equilibrated at room temperature. This starting temperature represents a typical gas temperature for CS, but does not take into consideration the cooling effects of the gas and particle after the gas expands through the divergent section of the de Laval nozzle during CS. The ability to investigate the evolution of temperature, and the role of impact velocity, is a critical test of this method, in addition to incorporating an accurate description of the atomic scale deformation physics of the particle and the substrate during CS impact. This manuscript demonstrates the ability of the QCGD method to model the impact behavior of powder particles and to resolve the roles of the deformation behaviors of the powder and the substrate that are likely precursors for the experimentally observed jetting and metallurgical bonding of the particles during CS particle impact at the experimental time and length scales of the process.

## Results

### Evolution of Morphology, Temperature and Pressure

**Figure 1:**
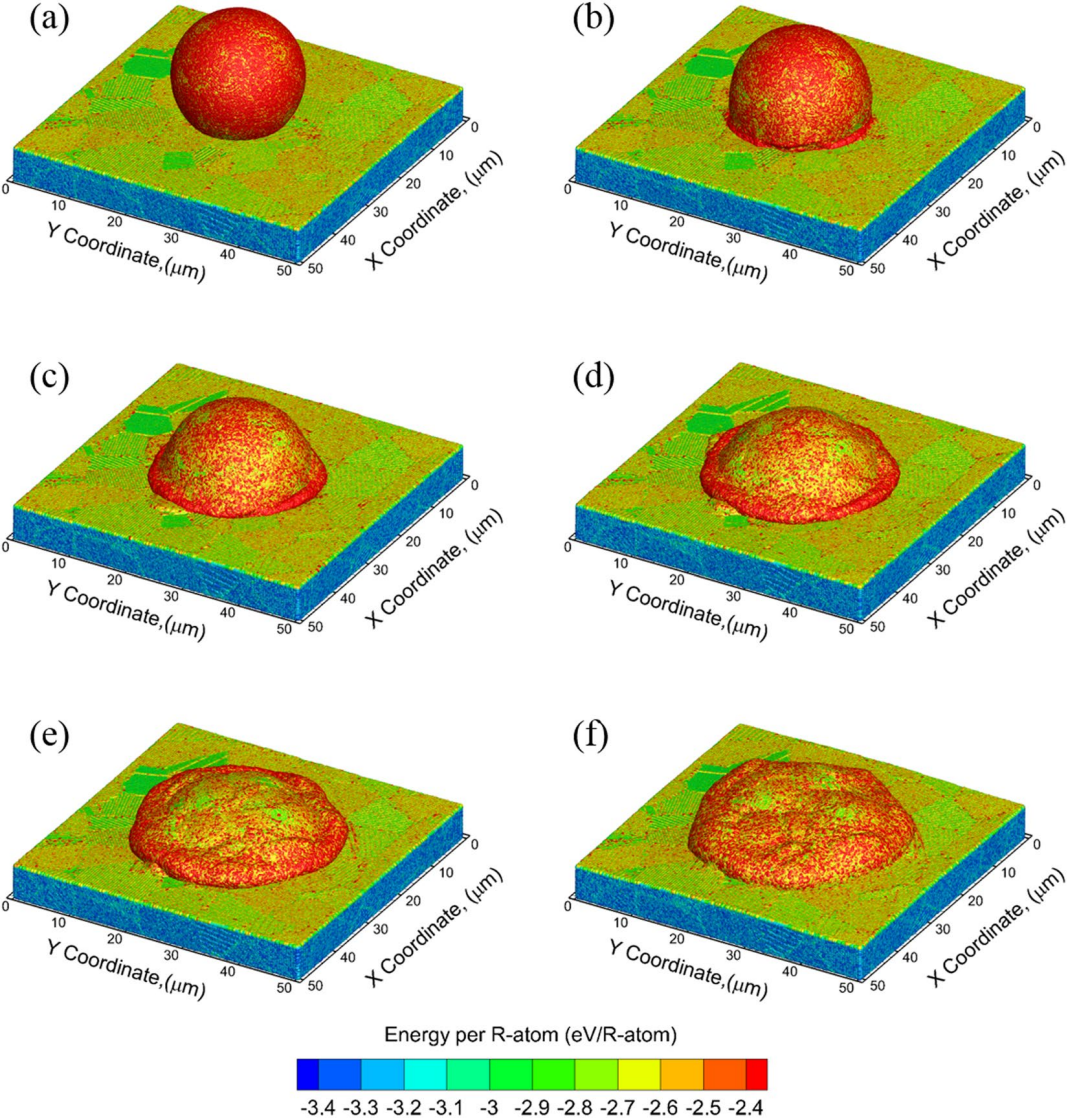
Splat morphology of a single particle impact model with an impact velocity *v*_*i*_ = 1600 m/s; snapshots at (**a**) t = 2 ns, (**b**) t = 4 ns, (**c**) t = 6 ns, (**d**) t = 8 ns, (**e**) t = 12 ns, and (**d**) t = 20 ns. The color of the atoms corresponds to the total energy of each R-atom in the QCGD simulations and is the same for all snapshots.

**Figure 2:**
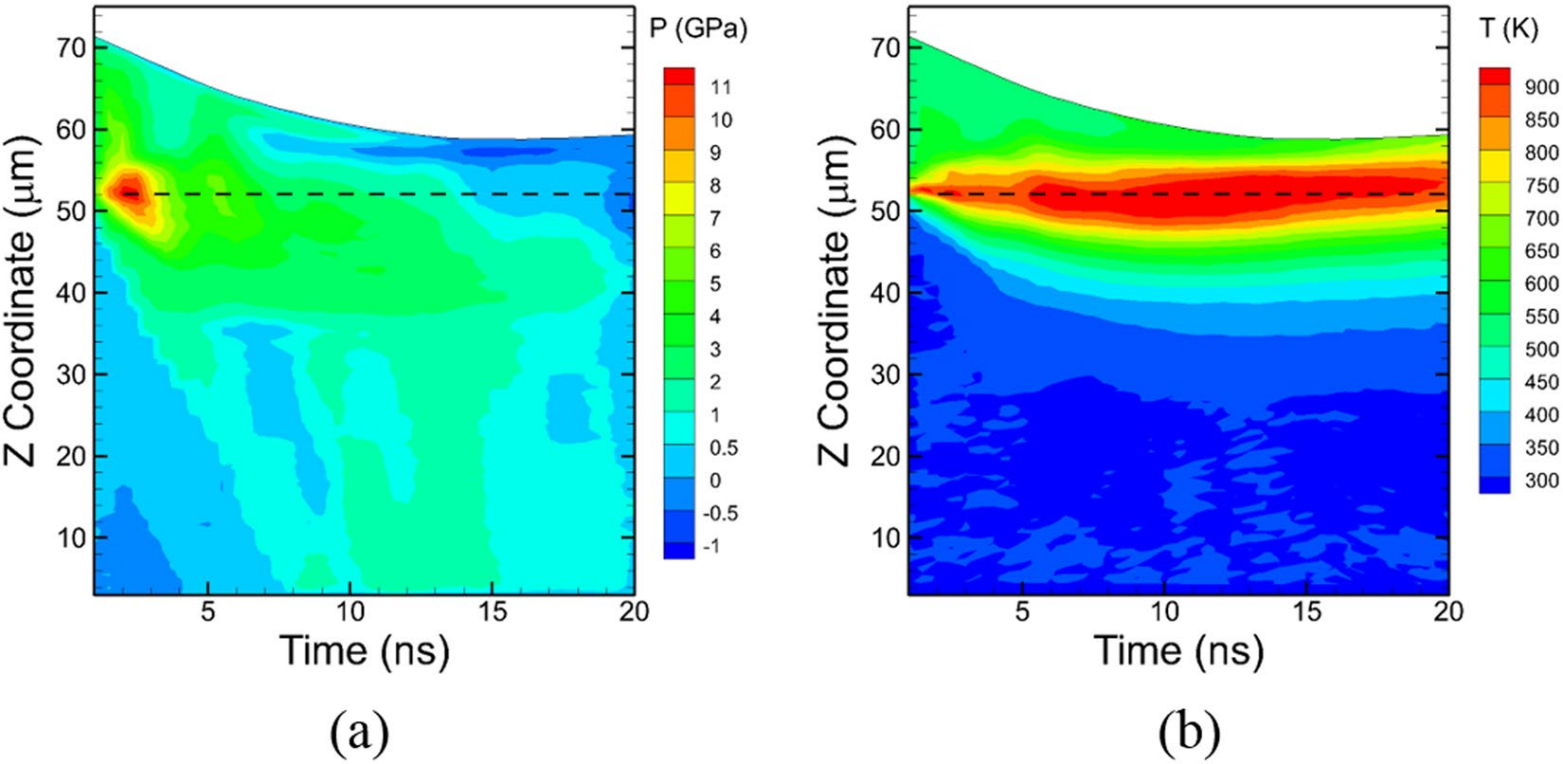
Evolution of average (**a**) pressure and (**b**) temperature generated in a thin vertical section through the center of the particle (for *v*_*i*_ = 1600 m/s) and the substrate along the Z axis (See Supplemental Information S1).

Illustrative snapshots showing the deformation behavior of a 20 µm polycrystalline Al powder particle at various times during impact at a velocity of 1600 m/s are shown in Fig. 1. The impact of the particle with the substrate occurs at a time of 2 ns as shown in Fig. 1(a), and an outward flow of the particle is observed as early as 4 ns, as shown in Fig. 1(b), when most of the particle is still accelerating towards the substrate. The continued penetration of the particle into the substrate enhances this outward flow behavior resulting in a thicker ring of metal from the impact region as shown in Fig. 1(c,d). The particle deformation is stabilized at ~12 ns as seen in Fig. 1(e), and the final morphology that results at ~20 ns is shown in Fig. 1(f). The temporal evolution of the average pressure and temperature generated in a cuboidal region central to the system along the impact direction (See Supplementary Note 5) are plotted in Fig. 2(a,b), respectively using a 5 µm × 5 µm thin section around the center of the system in the XY plane and along the impact axis (Z-axis). The dashed line indicates the original location of the particle/substrate interface. It can be seen that the impact generates a compressive pressure wave that reaches a maximum value of ~13 GPa and a shock wave that travels towards the bottom of the substrate as well as the top of the particle. The damping boundary conditions at the bottom of the substrate absorb this shock wave, whereas the shock wave in the particle reflects back from the top surface and creates tensile pressures (low) in the particle at a time of ~15 ns. The compressive pressures of the particle are dissipated by a time of ~20 ns, whereas the high temperatures at the interface due to the deformation of the particle and the substrate are dissipated by a time of ~50 ns. These times for the kinetic energy (pressure wave) dissipation are in excellent agreement with the times observed experimentally of less than 100 ns^5^.

### Role of Particle and Substrate Deformation

**Figure 3:**
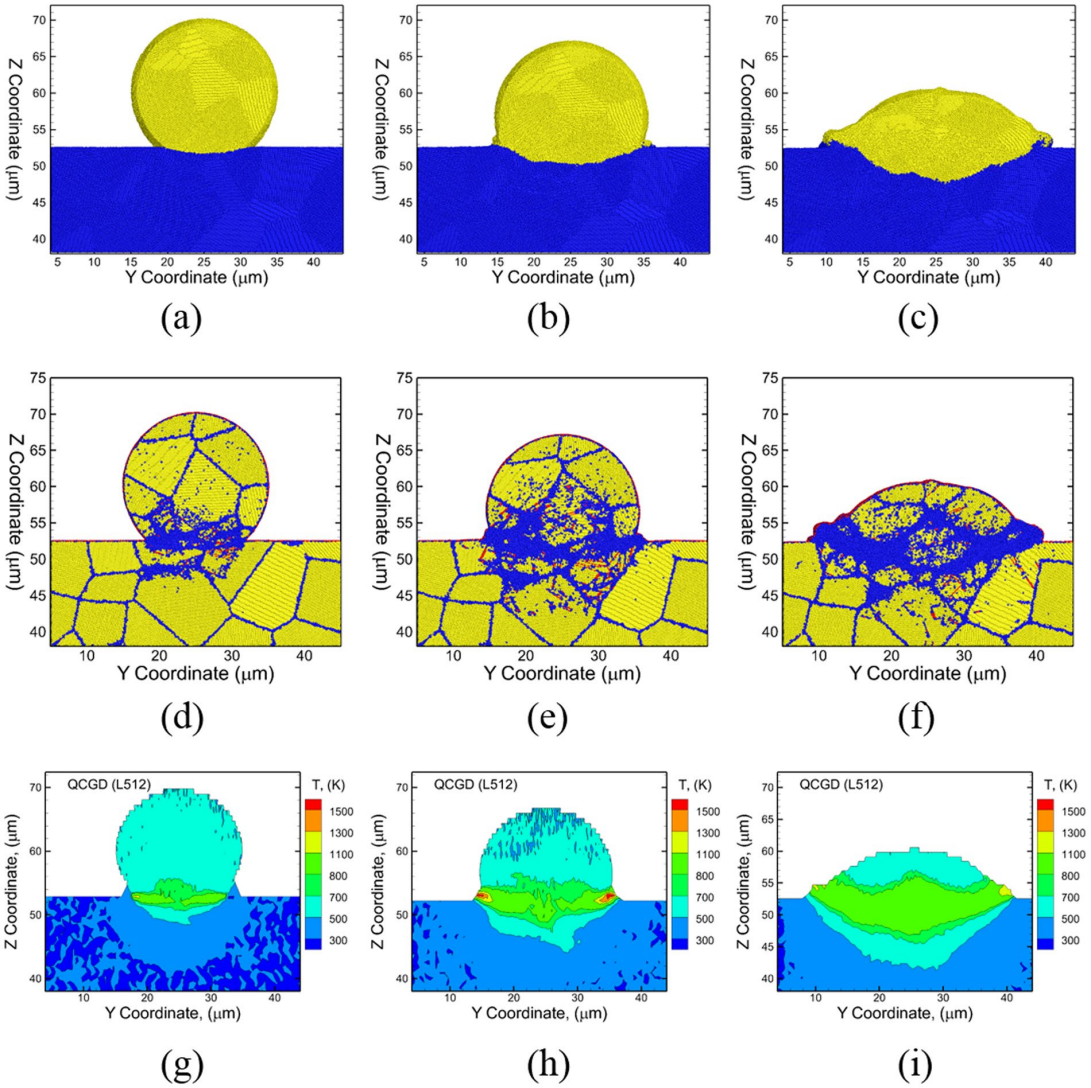
Snapshots showing the yellow R-atoms of the particle and blue R-atoms of the substrate during CS impact at a velocity of 1600 m/s at (**a**) t = 2 ns (just after impact), (**b**) t = 4 ns (visual evidence of “jetting”), and (**c**) t = 10 ns (maximum penetration depth reached). The corresponding plots showcasing the microstructural features are shown from (**d**–**f**). The atoms are colored based on common neighbor analysis and yellow atoms represent fcc stacking, red atoms represent hcp stacking, purple atoms represent a surface and blue atoms represent a disordered structure. The 2D plots showing the variations of temperatures at the times of (**d**) t = 2 ns showing the highest temperature zone generated at the center of the interface, (**e**) at t = 4 ns showing the shift of the peak temperatures to the periphery of the interface and corresponds to the onset of “jetting”, and (**f**) at t = 10 ns where heat transfer spreads high temperatures into the bulk of the particle due to continued deformation of the particle and the substrate.

**Figure 4:**
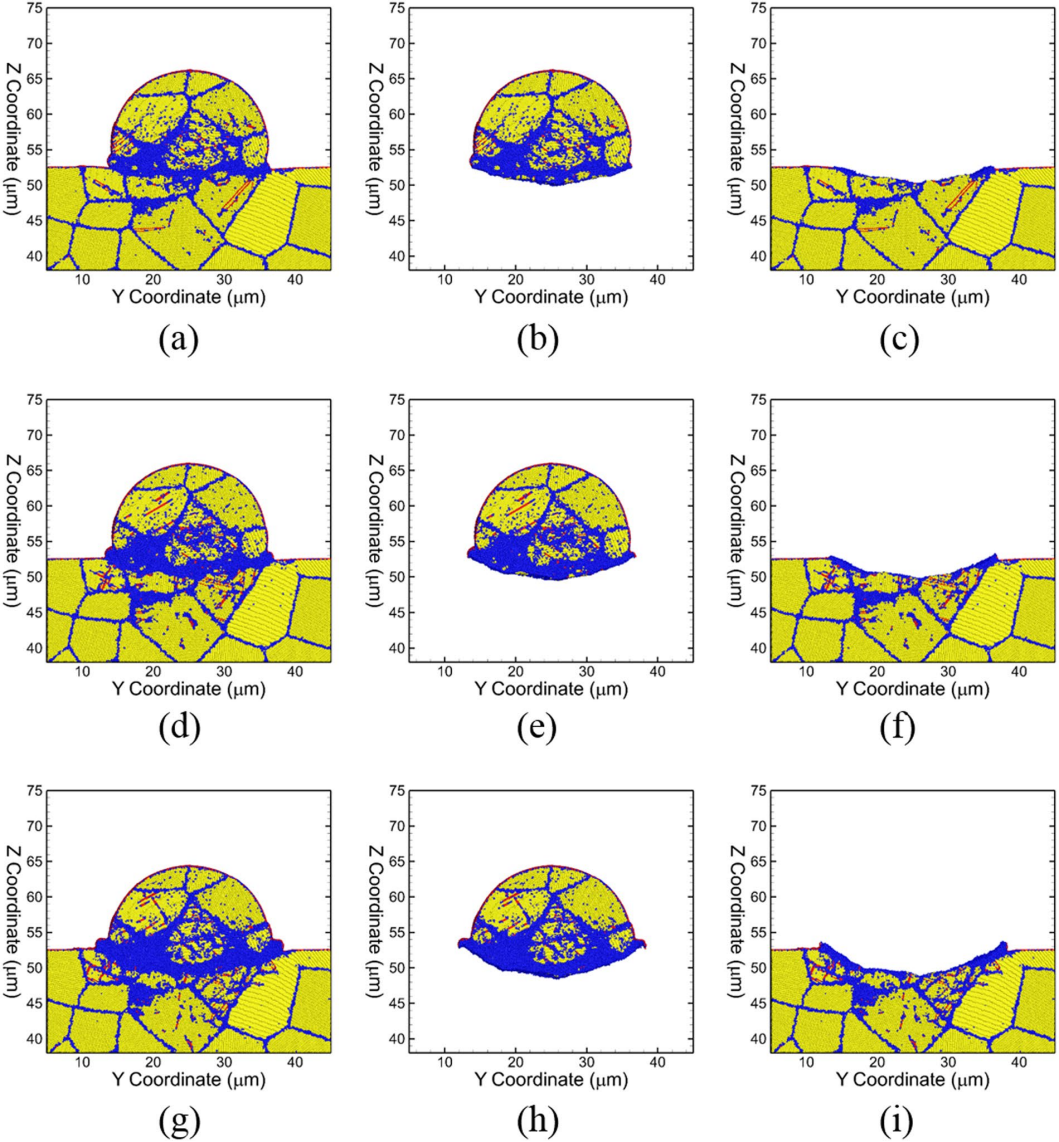
Snapshots showing microstructural features of (from L-R) the full system, particle and substrate respectively for velocities *v*_*i*_ = 1000 m/s at t = 8 ns (**a**–**c**), *v*_*i*_ = 1300 m/s at t = 6 ns (**d**–**f**) and *v*_*i*_ = 1600 m/s at t = 6 ns (**g**–**i**). The atom colors are the same as in Fig. 3.

To investigate the role of particle and substrate deformation and the related evolution of temperature along the particle/substrate interface, an 8 µm thin cross section is used along the X-axis and across the center of the splat (See Supplementary Note 5). Illustrative snapshots of this section showing the deformation behavior of the particle and substrate, and the evolution of the particle/substrate interface, are shown in Fig. 3(a–c). The corresponding snapshots showing microstructural features including grains and defects are shown in Fig. 3(d–f), and the plots showing the distributions of the temperatures generated at these times are shown in Fig. 3(g–i), respectively. One important aspect of the microstructural evolution post-impact is the substantial deformation at the particle/substrate interface. The evolution of defects including stacking faults and twins (colored red in Fig. 3(d–f)) is observed primarily in the substrate. The regions in the particle and in the substrate near the interface are populated with disordered R-atoms (colored blue in Fig. 3(d–f)), and this is due to the thermal softening effects created by localized high temperatures. The highest temperature zone is observed to be at the center of the interface at a time of 2 ns due to impact. Continued deformation results in the outward flow of the particle near the periphery as shown in Fig. 3(b), and the highest temperature zones are now present at the periphery of the interface at a time of 4 ns (~2 ns after impact). Further deformation, as the particle continues to penetrate into the substrate, results in the displacement of the substrate metal (blue R-atoms), which facilitates an outward/upward flow of the particle R-atoms (yellow). Further clarification on the contributions of the particle and the substrate to the microstructural evolution for three different impact velocities are shown in Fig. 4 using the same R-atom color coding as that shown in Fig. 3(d–f). In each case the particle and substrate are shown together (Fig. 4(a,d,g)) and with the particle (Fig. 4(b,e,h)) and substrate (Fig. 4(c,f,i)) separately. For an impact velocity of 1000 m/s, almost no upward/outward material flow is observed at the interface (Fig. 4(a–c)). While examining microstructures for higher impact velocities like 1300 m/s (Fig. 4(d–f)) and 1600 m/s (Fig. 4(g–i)), a few observations can be made. First, it is evident that the substrate initiates the “jet” shape and the particle follows that path as the system evolves with time. Secondly, it is clear that both thermal softening and defects play a role substrate jetting, as the jetted region at the periphery of the interface is initially populated by both planar defects as well as thermally softened disordered atoms.

**Figure 5:**
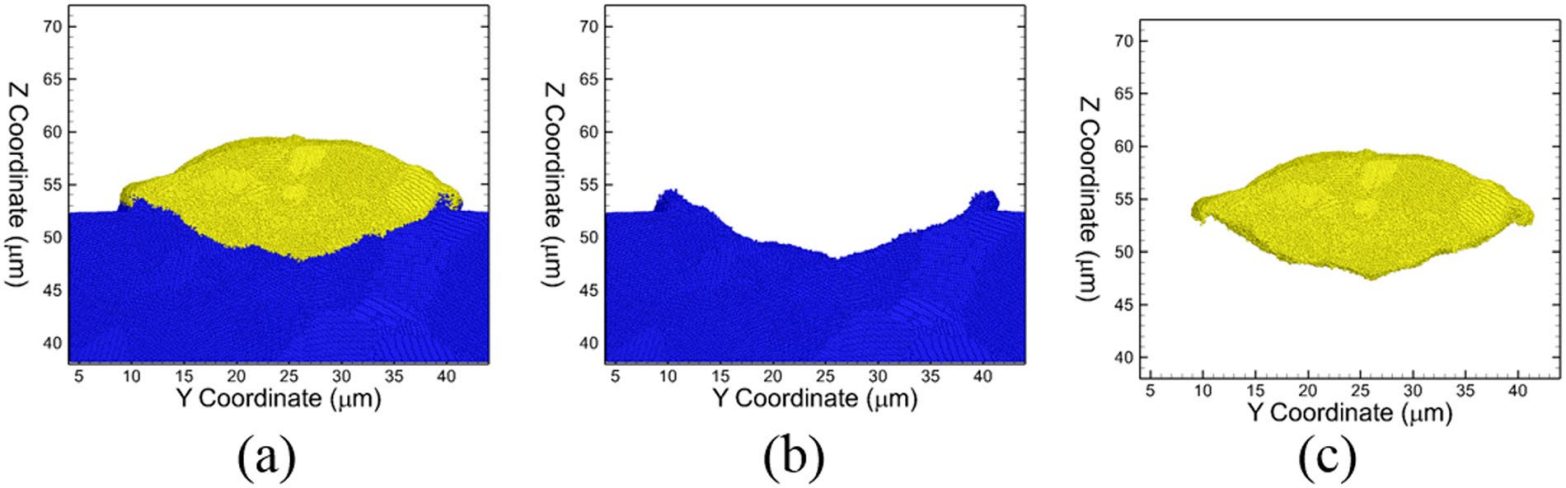
Snapshots indicating (**a**) the splat morphology of the Al particle at a time of t = 12 ns, (**b**) substrate displacement that initiated “jetting” and (**c**) particle morphology. The substrate R-atoms are colored blue and the particle R-atoms are colored yellow.

The temperature and the pressures in the region of the periphery predict the formation of a high temperature zone for a short time and lead to the final morphology, which is typical of an experimentally observed CS “splat”. Thus, the QCGD simulations reveal that material flow during particle impact occurs under the coupled effects of resistance of horizontal/vertical displacement of the substrate R-atoms and the residual translational kinetic energy in the upper region of the particle. In this case, the high temperature zone at the particle region near the periphery of the interface results in severe thermal softening of the particle. This is demonstrated by the sectional snapshots at a time of 12 ns as shown in Fig. 5(a) and the corresponding displacement of the particle (yellow R-atoms only) as shown in Fig. 5(b) and that of the substrate (blue R-atoms only) as shown in Fig. 5(c). In addition, the microstructural evolution is investigated to understand the thermal softening behavior of the particle at the interface (Supplementary Note 6). The heavily deformed regions of the particle and the substrate at the interface, as well as the high temperature regions at the periphery at a time of 12 ns, are observed to recrystallize by a time of 40 ns, and the final morphologies are typical of experimentally observed splats.

### Role of Particle Impact Velocity

**Figure 6:**
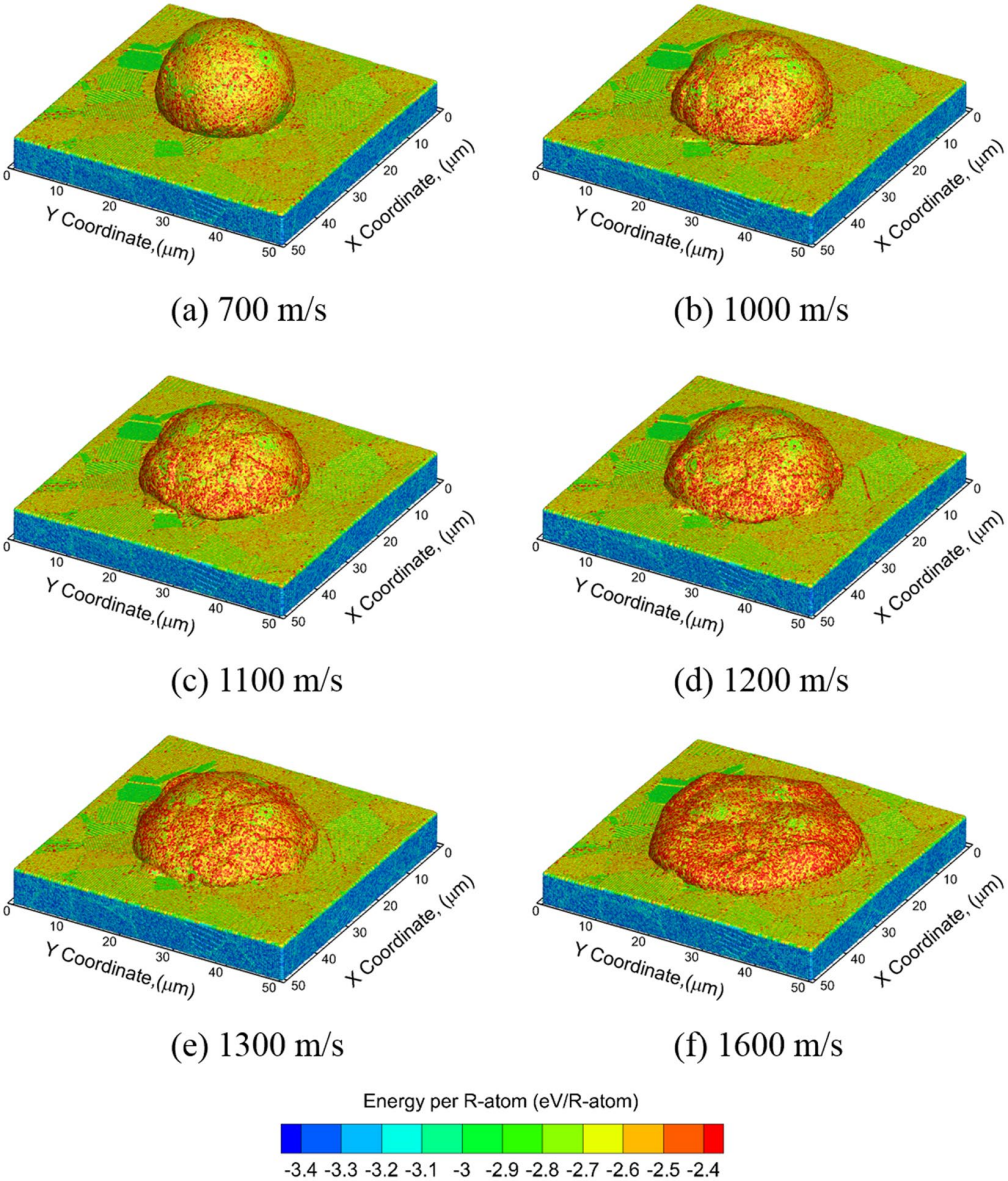
Final splat morphologies (at a time of t ~ 20 ns) at different impact velocities, (**a**) 700 m/s, (**b**) 1000 m/s, (**c**) 1100 m/s, (**d**) 1200 m/s, (**e**) 1300 m/s and (**f**) 1600 m/s.

The QCGD simulations are also carried out for various impact velocities and suggest that the evolution of the particle morphologies does not change after *t* = 40 ns for all cases considered here. Figure 6 shows the final morphologies of the “splat” formed for CS impact for different velocities perpendicular to the substrate at a time of *t* = 40 ns. The variations in morphologies are attributed to the larger amount of deformation in the particle and the substrate at higher impact velocities. The peak pressure values (at the center of the particle/substrate interface experiencing the impact) were calculated to be ~7 GPa, ~10 GPa, ~10 GPa, ~11 GPa, ~11 GPa and ~13 GPa for impact velocities of 700 m/s, 1000 m/s, 1100 m/s, 1200 m/s, 1300 m/s and 1600 m/s, respectively. More details of the pressure evolution for these impact velocities are provided in Supplementary Note 7. However, these snapshots do not reveal any information on the changes in the modes of deformation of the particle and the substrate for the range of velocities considered, as the model does not consider the presence of an oxide layer, and thus the contact of the particle with the substrate inevitably results in a metallurgical bond.

## Discussion

### Role of Thermal Softening

**Figure 7:**
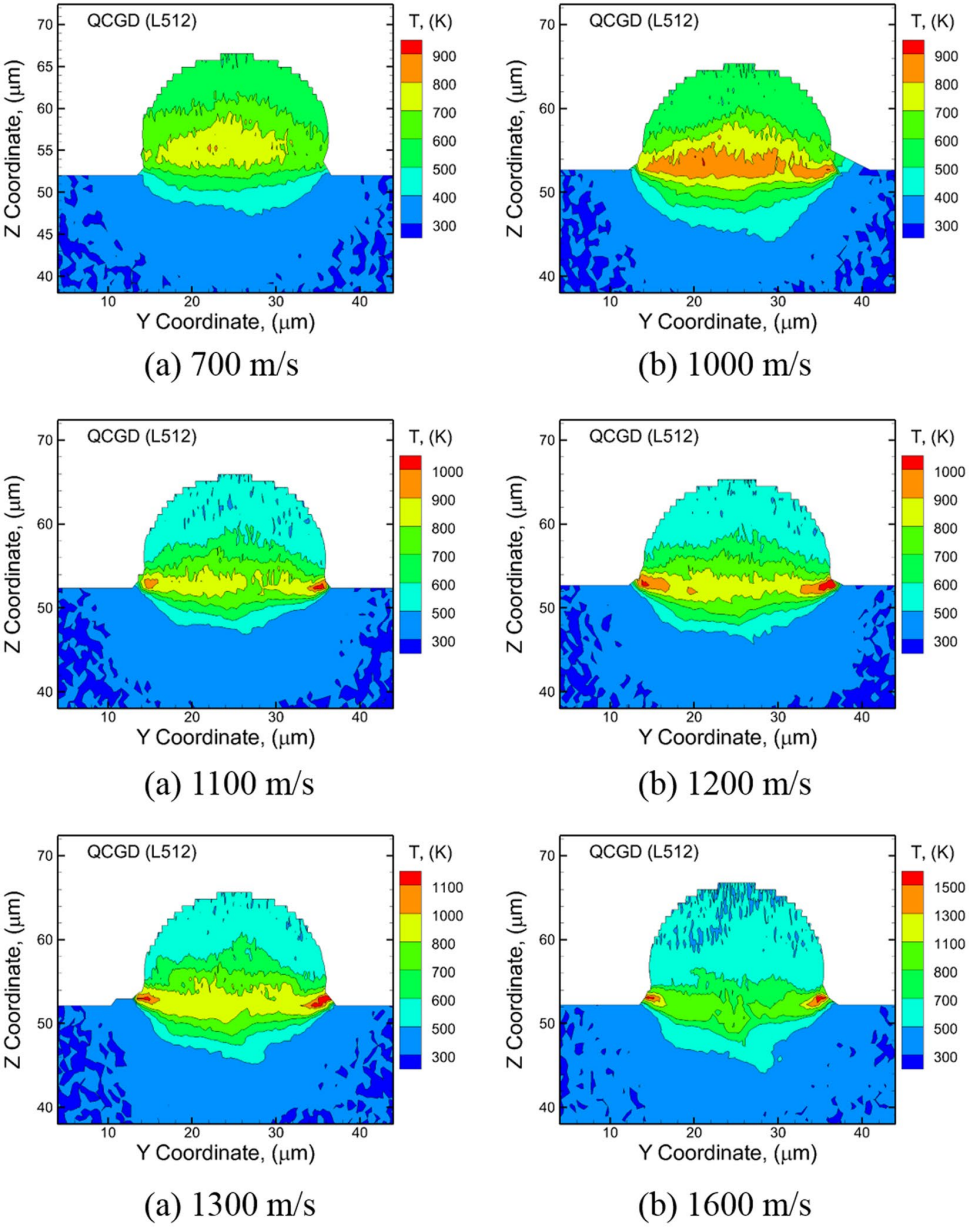
Snapshots at the time that showed the maximum temperature during the course of a full simulation for the cases of impact velocities of (**a**) 700 m/s, (**b**) 1000 m/s, (**c**) 1100 m/s, (**d**) 1200 m/s, (**e**) 1300 m/s and (**f**) 1600 m/s.

**Figure 8:**
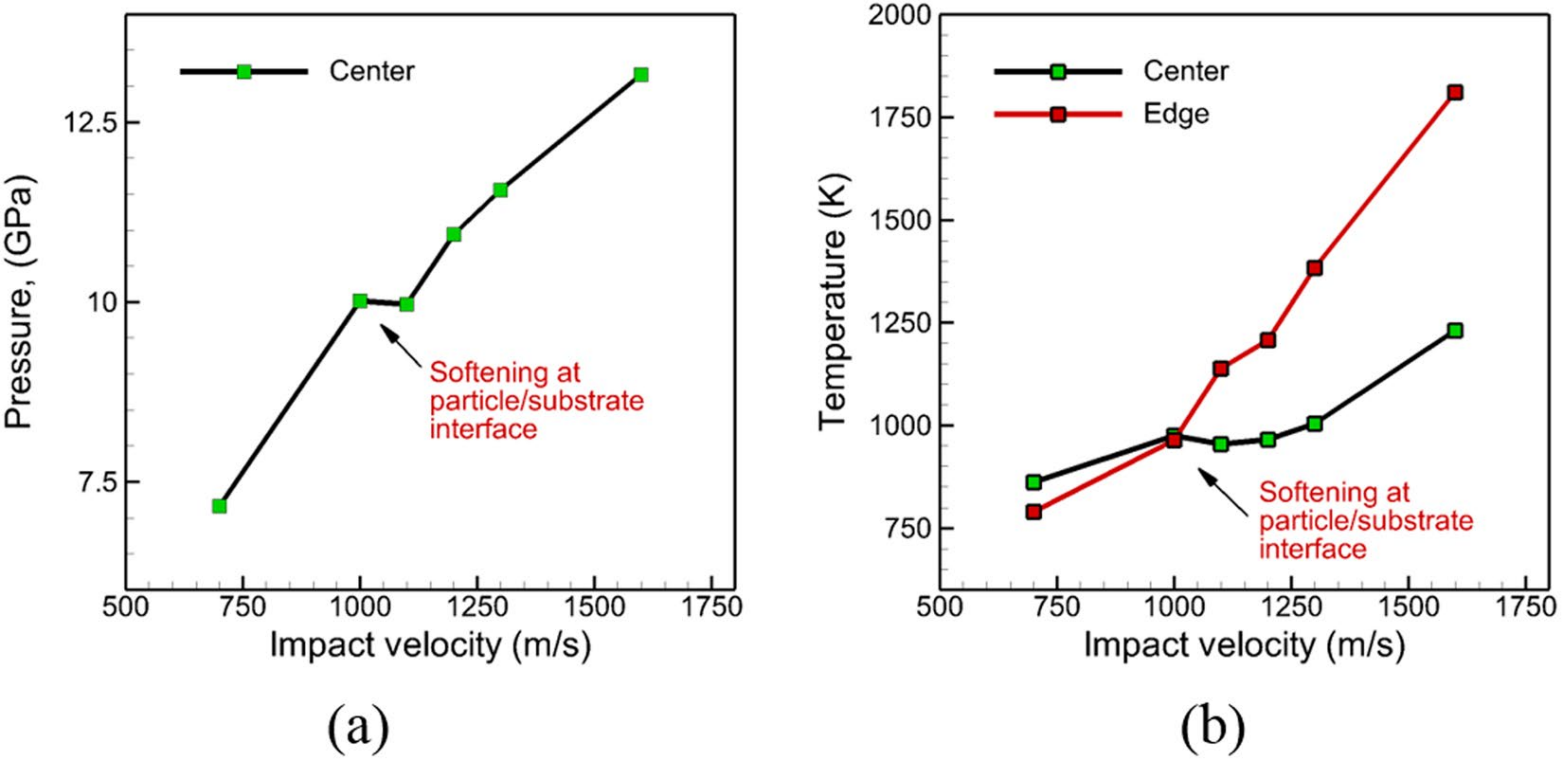
Plot of (**a**) peak pressure generated at the center of the particle/substrate interface at the location of impact, and (**b**) peak temperature generated at the center of the particle/substrate interface at the location of impact and at the periphery of the particle/substrate interface for different impact velocities considered here.

The changes in the modes of deformation with temperature can be observed through the predicted variations in the temperatures generated at the particle/substrate interface. The plots showing the distribution of the temperature along a thin section (as discussed before) at a time corresponding to a maximum in temperature post-impact are shown in Fig. 7 for the various impact velocities considered. A high-temperature zone is observed at the center of the particle/substrate interface for impact velocities of 1000 m/s and lower. For impact velocities above 1000 m/s, the high-temperature zone shifts from the center to the periphery of the particle/substrate interface. The computed values of the peak pressure generated at the center of the particle/substrate interface are plotted in Fig. 8(a) and the magnitudes of the peak temperatures generated along the particle/substrate interface are plotted in Fig. 8(b) for each of the impact velocities considered. The mode of deformation for impact velocities of 1000 m/s and below is restricted to regions close to the center of the interface, and the temperatures generated in this region are below the melting temperature of Al for an initial particle temperature of ~530 K before impact. As a result, the flow characteristics of the metal are largely limited, and the deformation behavior is determined by nucleation and evolution of dislocations. For impact velocities above 1000 m/s (as shown by the cross-over in the temperatures in Fig. 8(b)), the high-temperature zone shifts from the center to the periphery of the particle/substrate interface. This shift in the high temperature zones to the periphery, i.e. the softening of the material at the periphery, enables outward/upward flow of the particle and substrate, and is a likely precursor for the onset of jetting and metallurgical bonding observed experimentally during CS impact. The evolution of temperatures predicted here agrees very well with the experimental observation that no metallurgical bond is formed at the center of the impact region, and that bonding is largely localized at the periphery of the interface. In addition, due to the fact that the outward/upward flow is observed only for velocities greater than 1000 m/s, the velocity of 1000 m/s can be considered as a critical velocity for thermal softening at the periphery of the particle/substrate interface based on the QCGD simulations discussed here.

### Defining a Critical Velocity

Thus, the QCGD simulations allow us to unravel the microstructural evolution during the deformation of the particle and the substrate and the related evolution of temperatures during single particle impact. These simulations indicate that the initial outward flow occurs within 2 ns after impact, which contrasts with reported ASI at time scales of tens of nanoseconds after impact. The outward/upward flow of material at the particle/substrate interface observed here can be considered to be a precursor to the onset of “jetting” observed experimentally. However, none of the upward jetting observed experimentally occurs in the QCGD simulations. This deviation in jetting behavior is attributed to the immediate formation of a metallurgical bond in the QCGD simulations as no surface oxide layers are considered. A more accurate modeling of the jetting phenomena will require the incorporation of a surface oxide layer on the Al particles as well as a more accurate description of the particle temperatures prior to impact. It should be noted that the simulations discussed here assume particles equilibrated at the temperature of the gas stream. An accurate representation of the particle temperatures will need to incorporate the cooling of the particles as they are accelerated towards the substrate^6^.

Experimentally, a critical velocity has been identified for bonding of the powder particles during CS impact and the value of this critical velocity varies with the particle size, the particle temperature, the melting temperature of the material, and the oxide layer on the particle surface^13,29–31,64,65^. The critical velocity for thermal softening at the periphery of the particle/substrate interface evaluated here is slightly higher than the critical velocity of ~800 m/s defined experimentally for bonding of Al powder particles^65^ based on measurements of coefficient of restitution. Additionally, the EAM potential used here^60^ to define the scaled interactions between R-atoms over-predicts the melting temperature for Al by ~120 K, and this is likely to be the primary cause of the over-estimation of the critical velocity. The QCGD simulations also make approximations on the collective representations of the dynamics of atoms as well as the collective nucleation, interaction and evolution of dislocations. While the scaling-relationships for the QCGD simulations retain the collective description of the atomic scale mechanisms related to plastic deformation (nucleation of dislocations; interactions of dislocations), a slight strengthening of the metal is observed when compared to a similar system simulated using classical MD^63^, and hence this effect can lead to a modest over-prediction of the impact velocities needed for the onset of jetting.

It should be noted that the critical velocity estimated here is based on impact of pure Al particles equilibrated at the heated gas temperatures onto pure Al substrates. While these simulations demonstrate the ability to model single particle CS impact, a more realistic modeling of the CS impact will require the incorporation of more accurate temperatures (cooling effects of the particle) prior to impact. In addition, the deformation behavior and the evolution of temperature observed here is likely to be affected by particle microstructures (grain size, distribution and size of alloying phases, surface oxide layer), impact velocities and particle temperatures prior to impact. As a result, a direct comparison with experimental observations and investigation of bonding mechanisms requires a more accurate representation of the particle microstructures with distribution of grain sizes, pores, alloying phases and an oxide layer as well as substrate microstructures in addition to particle shapes, particle temperatures and impact velocities.

In conclusion, large scale QCGD simulations of single particle impacts are carried out for a 20 µm polycrystalline Al particle on to a polycrystalline Al substrate and suggest that the deformation behavior and the evolution of microstructure and temperature is determined by the impact velocity for a given particle size. A critical velocity for thermal softening at the periphery of the particle/substrate interface is identified to be ~1000 m/s for an Al particle size of 20 microns by the QCGD method using scaling relationships for the EAM potential based on the evolution of microstructure and temperatures. The outward flow is first observed in the particle followed by extensive deformation and upward flow of the substrate material in contact with the particle. The deformation of the particle and substrate results in high temperature zones at the periphery of the particle/substrate interface. Thus, the QCGD simulations enable us to unravel the roles of particle and substrate deformation that lead to localized softening of the metal at the periphery of the particle/substrate interface. The resultant outward/upward flow of the particle and substrate is a likely precursor for the experimentally observed jetting and hence bonding of the particles during CS impact. These results demonstrate the capability of the mesoscale QCGD method to unravel the microstructural evolution during the deformation of the particle and the substrate and the evolution of temperatures (and thermal softening behavior) during CS impact at the experimental time and length scales.

## Materials and Method

### The Quasi-Coarse-Grained Dynamics (QCGD) Method

The QCGD method^59^ is a mesoscale modeling method that uses a coarse-grained (CG) representation of atomic scale microstructure and representative atoms (R-atoms) to define the collective dynamics of several atoms in an atomistic microstructure. The scaling relationships used here consist of a coarse-grained representation of a volume of atomistic unit cells of Al by 1 CG unit cell with 4 R-atoms. The scaling relationships need to be defined for the atomic scale interatomic potentials to retain the MD-predicted energetics for the R-atoms in the CG microstructure. The scaling relationships and correspond to a “distance scaling parameter” of *A*_*cg*_ = *n* and a “number of atoms represented” parameter of *N*_*cg*_ = *n* × *n* × *n* that scales the degrees of freedom of the R-atoms to account for the missing atoms in the microstructure. The scaling parameters are used to scale the EAM interatomic potential for Al as well as the degrees of freedom to reproduce the equation of state (EOS) and the high temperature behavior of Al as calculated using MD simulations^59^. The QCGD simulations are able to reproduce the thermodynamic behavior (melting, phase transformation, etc.) and mechanical behavior (plastic deformation and failure) at high strain rates as well as under shock loading conditions for FCC metals^59^ as well as HCP metals^61^. The scaling relationships and the QCGD framework retain the atomic scale characteristics of the energetics and dynamics (nucleation, interactions, and reactions) of collective dislocations as predicted in MD simulations. These include the descriptions of the kinetics and densities of the relative fractions of nucleation, interaction mechanisms, dissociation, and recombination mechanisms observed in MD simulations under conditions of shock loading^63^. The QCGD scaling relationships can be defined for any level of coarsening and have been validated to reproduce the experimentally observed shock deformation and spall strengths at length scales of tens of microns retaining the atomic level deformation physics of the processes involved. The capability of the various levels of coarsening for the QCGD simulations to retain the atomistic characteristics of the deformation behavior (from MD simulations) during impact/shock is discussed in^62^. The framework of the QCGD simulations and the capability of L512 scaling relationships is discussed briefly in Supplementary Note 1. The ability of the QCGD simulations to retain the MD-predicted atomic scale mechanisms of defect nucleation and evolution, shock wave propagation and reflection, and temperature evolution during single particle impact using scaling relationships for the atomic scale interatomic potentials and the degrees of freedom is demonstrated in Supplementary Note 2 and Supplementary Figs 1 and 2. The QCGD simulations are carried out using L512-scaling of the EAM interatomic potential for Al^60^ and degrees of freedom and can be considered as a particle-based method or a discrete element method. The L512 scaling relationships are able to reproduce the MD-predicted equation of state (EOS) as well as the pressure and temperature dependence of the energy of a volume of EAM Al as shown in Supplementary Fig. S3 and discussed in Supplementary Note 3.

### Cold Spray Single Particle Impact

In order to demonstrate this approach for modeling CS, QCGD simulations are used to investigate the impact behavior of a single polycrystalline Al powder particle with a diameter of 20 microns onto a polycrystalline Al substrate under conditions observed in CS experiments. An average grain size of ~10 µm is used for both the particle and the substrate as a first approximation of the model systems. The 20 µm diameter polycrystalline Al particle modeled using ~2 million R-atoms is impacted onto a 50 µm × 50 µm × 50 µm polycrystalline Al substrate modeled using ~55 million R-atoms at impact velocities ranging from 700 m/s to 1600 m/s perpendicular to the substrate (Z direction). Periodic boundary conditions are used in the X and Y directions and the simulations are carried out for a duration of 50 ns with a time step of 0.5 ps. More details of the CS setup used to model single-particle impact of the Al particles are provided in Supplementary Note 4 and shown in Supplementary Fig. S4. As a first approximation, the particle (yellow) is equilibrated at a temperature of ~530 K of a heated gas stream, and the substrate is equilibrated at room temperature. This temperature represents the starting gas temperature but does not take into consideration the cooling effects of the gas and particle after the gas expands through the diverging section of the de Laval nozzle. The particle is then accelerated towards the substrate at various impact velocities. A thin rigid layer (light green) is maintained at the bottom of the substrate to prevent substrate shift in the direction of impact (z-direction). In addition, damping is also used at the boundary regions (light blue) to absorb the incoming impact-induced pressure wave to negate the artifacts that could arise due to reflection of the pressure wave off the boundary surfaces.

### Polycrystalline Microstructures

The “*Voronoi construction method*”^66^ is used to build the initial microstructures with periodic lateral directions (X, Y) and free in the loading direction (Z).

### Interatomic Potentials

The interactions between the R-atoms are based on scaling relationships for the “embedded atom method” (EAM) potential for Al^60^.

## Electronic supplementary material

SUPPLEMENTAL INFORMATION
