# Supplementary material for: Unraveling the Mesoscale Evolution of Microstructure during Supersonic Impact of Aluminum Powder Particles
Source: Sci Rep. 2018 Jul 4;8:10075. doi: 10.1038/s41598-018-28437-3 (PMC6031617; doi:10.1038/s41598-018-28437-3)
Supplement: Supplementary file 1 — SUPPLEMENTAL INFORMATION [file 41598_2018_28437_MOESM1_ESM.docx]

**Supplementary Information**

**Unraveling the Mesoscale Evolution of Microstructure during Supersonic Impact of Aluminum Powder Particles**

**Authors:** Sumit Suresh^1^, Seok-Woo Lee^1^, Mark Aindow^1^, Harold D. Brody^1^, Victor K. Champagne Jr.^2^, and Avinash. M. Dongare^1,*^

**Affiliation:**

1. *Department of Materials Science and Engineering, Institute of Materials Science, University of Connecticut, 97 North Eagleville Road, Storrs, CT 06269, USA*
2. *U.S. Army Research Laboratory, Weapons and Materials Research Directorate, Aberdeen Proving Ground, Aberdeen, MD 21005, USA.*

* **Corresponding Author:**

Avinash M. Dongare

- **Address)** Department of Materials Science and Engineering & Institute of Materials Science, 97 North Eagleville Road, Unit 3136 Storrs, CT 06269-3136, USA
- **Email)** [dongare@uconn.edu](mailto:dongare@uconn.edu)
- **Telephone)** +1 860-486-2592

**Supplementary Note 1: Scaling Relationships for QCGD Simulations**

For a metallic system, the energy of an atom in MD simulations is calculated using the embedded atom method (EAM) potential [[[1]](#endnote-1)] as

$E_{i}=\frac{1}{2}\sum_{j\neq i} \varphi_{ij}(r_{ij})+F_{i}\left(\sum_{j\neq i} f_{j}(r_{ij})\right)$ (1)

Here $r_{ij}$ is the distance between neighboring atoms; is the pair energy term defined as a function of the interatomic distance; and $F_{i}$ is the embedding energy term defined as a function of the electron density at the position of atom. The energy values for the R-atoms in the QCGD simulations are then defined by scaling the interatomic potential by a distance scaling parameter ($A_{cg}$) to retain the energy and force values as would be predicted in the MD simulation [[[2]](#endnote-2)]. This scales the equilibrium spacing between the R-atoms and the cutoff distance by$A_{cg}$. The energy of the R-atom is then defined as

(2)

Here, $r_{ij}^{R}$ is the interatomic distance between R-atoms. The forces on each R-atom are therefore the same as would be predicted in the MD simulations. Thus, for the representation of $n\times n\times n$ unit cells of an atomistic system by one CG-cell, $A_{cg}$is set to be equal to the number of unit cells ‘*n*’ being represented in each direction. For example, $A_{cg}$ corresponds to a value of 2 for the L2-scaling relationships. The representation of $n\times n\times n$ unit cells of an atomistic system by one CG-cell implies that the dynamics of each R-atom represents the average dynamics of several atoms (For example, 8 atoms when one FCC CG-cell represents $2\times2\times2$ atomic FCC unit cells).

The number of atoms represented by the R-atom need to be accounted for to define the total energetics of the atomistic system as would be predicted in a MD simulation. The total potential energy for the CG-system is reproduced by scaling the potential energy of the R-atoms by the number of atoms being represented by each R-atom to account for all the atoms represented by the R-atom. This scaling parameter is defined to be $N_{cg}=n\times n\times n$ for the representation of $n\times n\times n$ unit cells of an atomistic system by one CG-cell. The velocities for each R-atom and the kinetic energies are calculated in the same way as would be calculated for atoms in the MD simulation. The scaling relationships also allow for increased time-steps for the QCGD simulations. The elements of the atomic-level stress tensor for the R-atom are calculated as

$\sigma_{\alpha\beta}^{\text{R}}\left(\text{i}\right)=-\frac{1}{\text{Ω}_{0}}\left[\frac{1}{2}\sum_{\text{j}} \text{F}_{\text{ij}}^{\alpha}\left(\frac{\text{r}_{\text{ij}}^{\beta}}{A_{cg}}\right)+m_{i}^{R}v_{i}^{R\alpha}v_{i}^{R\beta}\right]$ (3)

Here, α and β label the Cartesian components, where Ω_0_ is the atomic volume for the R-atom, F_ij_ is the force on atom *i* due to atom *j*, $m_{i}^{R}$ is the mass of R-atom *i*, and $v_{i}^{R}$ is the velocity of R-atom *i*. Thus, the scaling of the interatomic potentials retains the energetics for the individual R-atoms for any strain state, or temperature or pressure in the CG-configuration as would be predicted using MD simulations. More details about the QCGD framework, capabilities and limitations can be found in Reference [2].

The capability of the higher levels of coarsening to reproduce plasticity mechanisms is has been demonstrated in Reference [[[3]](#endnote-3)] under shock loading conditions. An exact comparison of these defect density fractions between MD vs L2-QCGD, L2-QCGD vs L4-QCGD, L4-QCGD vs L8-QCGD, L8-QCGD vs L16-QCGD, L16-QCGD vs L32-QCGD, and L32-QCGD vs L64-QCGD simulations is used to test the capability of the various levels of coarsening to model the plastic deformation behavior and spall failure behavior. The reference demonstrates that the QCGD method retains the capability of the scaling relationships to model the atomic scale characteristics (Burger’s vectors and contributions to plasticity) related to nucleation, interaction and evolution of the various types of dislocations under shock loading and spall failure irrespective of the level of coarsening. The validity of any level of coarsening is observed to be determined by the ability of L512 scaling relationships to reproduce the equation of state (EOS) and the high temperature behavior of Al as predicted using MD simulations. A direct comparison of the grain growth has not been investigated by QCGD simulations, but all the simulations discussed in [62] comprise of polycrystalline microstructures. It should be noted that since the QCGD models the collective evolution of defects, the energetics of all the nucleation, interaction and evolution mechanisms is scaled to account for the collective nature of the phenomena and can render scaling relationships to predict atomistic values based on values predicted using QCGD simulations. The QCGD method therefore scales up the capabilities of MD simulations to bridge the gap at the mesoscales. All these details are now included in the revised version of the manuscript.

**Supplementary Note 2: L2-QCGD vs MD Single Particle Impact**

The capability of the QCGD method to model single particle impact is demonstrated for a single crystal Al particle with a diameter of 75 nm equilibrated at a temperature of 600 K. The Al particle is impacted on to a rigid substrate using a velocity of 1000 m/s using MD and L2-QCGD simulations. The MD and QCGD systems built to model this impact comprised of ~16 Million and ~2 Million atoms/R-atoms, respectively. The L2-scaling indicates a CG representation of a volume of $2\times2\times2$ atomistic unit cells of Al by 4 R-atoms and corresponds to a “distance scaling parameter” of $A_{cg}$= 2 and a “number of atoms represented” parameters of $N_{cg}$= 8. It should be noted that an L2-QCGD R-atom will account for the dynamics of 2^3^, i.e. 8 regular atoms. The snapshots shown in Supplementary Figure 1 compare the temperature evolution of the particle deformation at different times as predicted by the MD and L2-QCGD simulations. It can be seen that the L2-QCGD simulations retain the MD predicted dynamic evolution of temperature in the particle attributed to plastic deformation of the particle during impact. A comparison of the final splat morphologies in Supplementary Figure 2 predicted by the QCGD and MD simulation demonstrates the capability to model the dynamic evolution of microstructure during single particle impact using a significantly reduced number of R-atoms and retaining the atomic scale mechanisms of defect nucleation and evolution, shock wave propagation and reflection, and temperature evolution and scaling relationships for the atomic scale interatomic potentials and the degrees of freedom.

**Supplementary Note 3: L512-QCGD Scaling relationships**

The L512-scaling relationships used here consist of a coarse-grained representation of a volume of $512\times512\times512$ atomistic unit cells of Al by 4 R-atoms and correspond to a “distance scaling parameter” of $A_{cg}$= 512 and a “number of atoms represented” parameter of $N_{cg}$= 134,217,728. These scaling parameters are used to scale the EAM interatomic potential for Al as well as the degrees of freedom to reproduce the equation of state (EOS) and the high temperature behavior of Al as predicted using MD simulations. The L512 scaling relationships for the simulations discussed here allow a time step of 0.5 ps that, not only render energy conservation in the NVE ensemble, but also are able to reproduce the shock pressures and the decay of the elastic precursor as obtained for a time step of 0.002 fs. The L512 scaling relationships are able to reproduce the MD-predicted equation of state (EOS) as well as pressure and temperature dependence of the energy of a volume of Al as shown in Supplementary Figure 3.

**Supplementary Note 4: Setup for Cold Spray Impact**

The QCGD simulations of single particle impact are carried out by accelerating a 20 µm diameter polycrystalline Al particle modeled using ~2 million R-atoms onto a 50 µm x 50 µm x 50 µm polycrystalline Al substrate modeled using ~55 million R-atoms at impact velocities ranging from 700 m/s to 1600 m/s perpendicular to the substrate (Z direction). Periodic boundary conditions are used in the X and Y directions and the simulations are carried out for 50 ns with a time step of 0.5 ps. Supplementary Figure 4 shows the CS setup used to model single-particle impact of the Al particles. A thin rigid layer (light green) is maintained at the bottom of the substrate to prevent substrate shift in the direction of impact (z-direction). In addition, damping is also used at the boundary regions (light blue) to absorb the incoming impact-induced pressure wave to negate the artifacts that could arise due to reflection of the pressure wave off the boundary surfaces.

**Supplementary Note 5: Pressure and Temperature Analysis**

To analyze the temporal response of the pressures generated in the system post impact, a 5 µm x 5 µm thin section, as shown in Supplementary Figure 5(a), was taken along the center of the “splat” and this system was divided into 80 bins along the impact axis (Z-axis). A similar analysis was constructed for the temperature evolution except that it was two dimensional for visual clarity and for observing the temperature evolution near the “jet” region. For the case of temperature analysis an 8 µm thin cross section along the X-axis was taken along the center of the splat as shown in Supplementary Figure 5(b) and the section was divided into 6400 bins in the YZ plane (80 bins each in Y and Z directions). For both analyses, the pressures and velocities were averaged for the whole bin and corrections for average center-of-mass velocity of the bin were accommodated to compute temperatures accurately.

**Supplementary Note 6: Microstructural evolution for impact velocity of 1600 m/s**

The impact simulation was run for a total duration of 50 ns after imparting an instantaneous velocity to the particle. An illustrative snapshot of the system at t=0 is shown in Supplementary Figure 6(a). For the case of *v_i_*=1600 m/s, the “jetting” behavior was observed at t=4 ns, according to the insights gained from visual analysis at the interface and the temperature contour plots. From Supplementary Figure 6(b), it can be seen that there is an overabundance of disordered (blue colored) atoms at the particle/substrate interface along with several stacking faults (red colored) in the bulk of both particle and the substrate. The color scheme here depicts any atom of a non-crystalline nature as disordered. Thus, the grain boundaries and regions of melting (which is confirmed to be the periphery of the substrate/particle interface from the temperature contour plots) are represented by the same color. Morphological stability was achieved after around t=12 ns as observed in Supplementary Figure 6(c), i.e., the penetration depth and overall splat dimensions were relatively unaffected subsequently. Prior to this, most of the severe plastic deformation and heat generation had taken place. The behavior that follows is heat dissipation towards the bulk of the particle and substrate, as is noticed in the temperature plots, and some elastic rebounding. Towards the end of the simulation run, there is also evidence of recrystallization as observed in Supplementary Figure 6(d), where most of the atoms that were of the disordered kind, now show fcc crystalline nature (yellow colored). The elastic rebound mechanism mentioned before leads to an overall flatter appearance at the top of the substrate and this is evident when comparing observed in Supplementary Figure 6(c) and (d).

**Supplementary Note 7: Pressure Evolution for Various Impact Velocities**

For all the cases of impact velocities studied, it was observed that a compressive pressure wave is generated just post impact and this is when it reaches its maximum value. The peak pressure values were calculated to be around 7 GPa, 10 GPa, 10 GPa, 11 GPa, 11 GPa and 13 GPa for the cases of 700 m/s, 1000 m/s, 1100 m/s, 1200 m/s, 1300 m/s and 1600 m/s respectively. Supplementary Figure 7(a)-(f) shows the different pressure profiles generated along the impact direction for the impact velocities included in this study. As expected, the compressive pressure wave generated post impact travels the depth of the substrate and slowly wanes away. Naturally, the time taken for this pressure to dissipate showed an increasing trend with the impact velocity. In general, around 20 ns of the simulation time was enough for the compressive pressure wave to dissipate for all cases. A tensile region was also found to form in the upper half of the particle region and this could be directly related to the final penetration depth of the particle in each case. This tensile region is most noticeable for lower impact velocities and its effect dies down as we increase the impact velocity. This tensile region could explain the low penetration observed for the 700 m/s case as seen in Supplementary Figure 7(a) because more regions of the particle favor an elastic rebound over the severe plastic deformation that is generally seen for other cases.


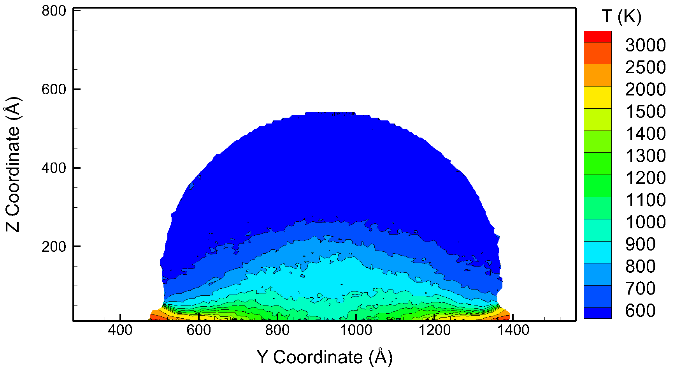

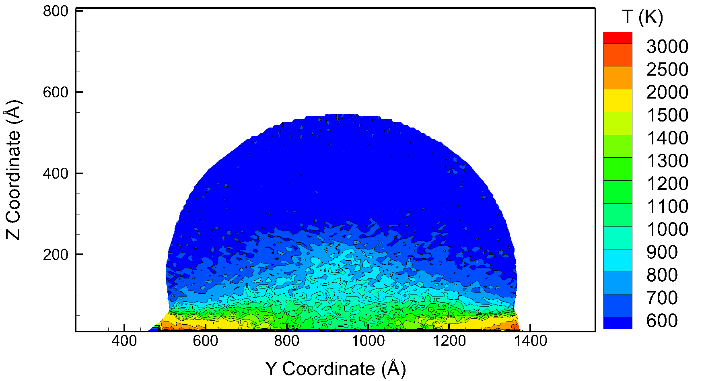


(a) MD (30 ps) (b) L2-QCGD (30 ps)


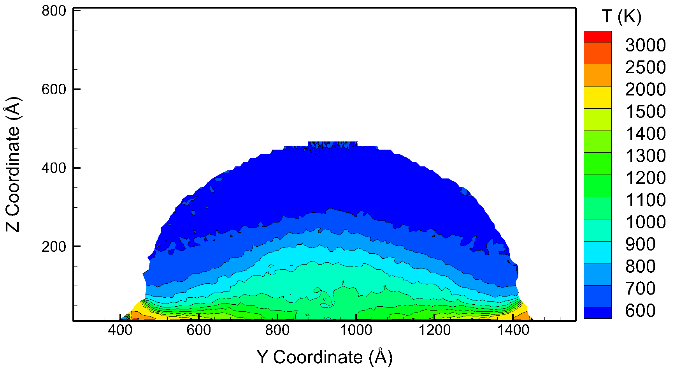

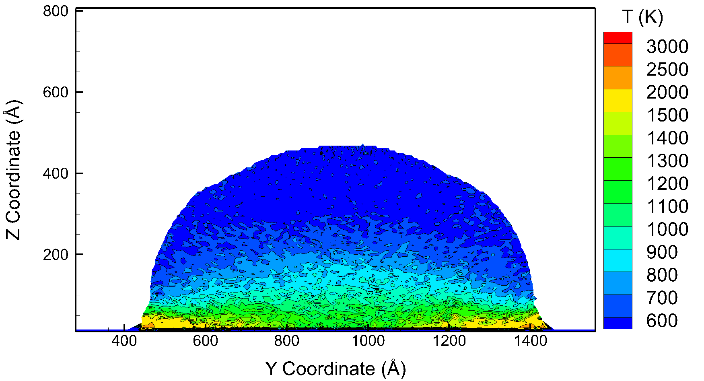


(c) MD (40 ps) (d) L2-QCGD (40 ps)


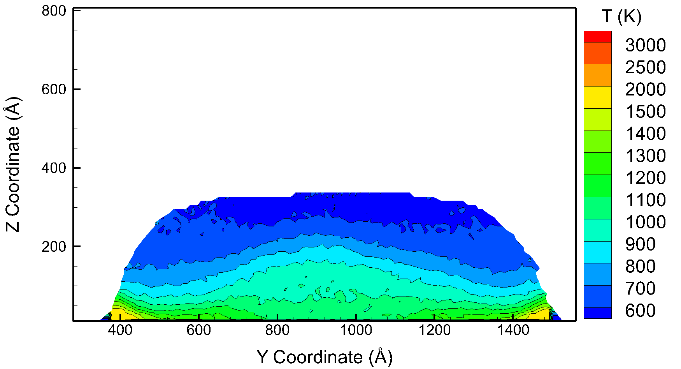

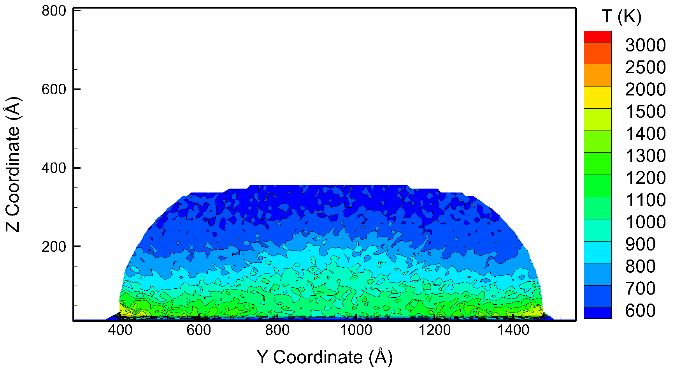


(e) MD (60 ps) (f) L2-QCGD (60 ps)

**Supplementary Figure S1: MD and QCGD comparison of single particle impact.** Comparison of the temperature contour plots between MD (left) and L2-QCGD (right) at a time of t=30 ps is shown in (a) and (b), respectively; at a time of 40 ps is shown in (c) and (d), respectively; and at a time of 60 ps is shown in (e) and (f), respectively.

**
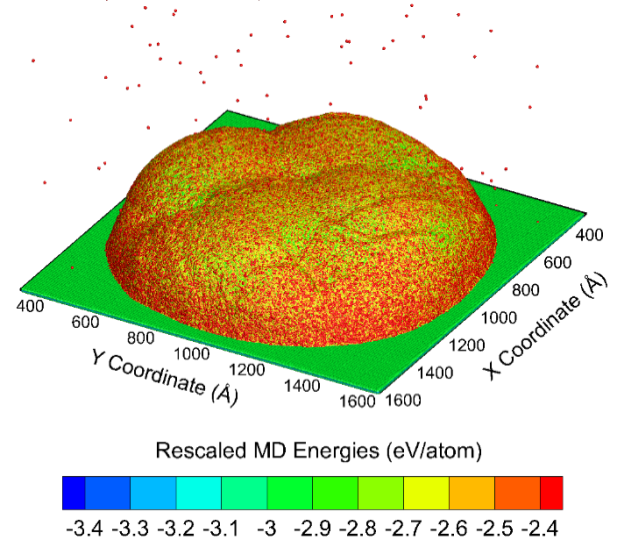

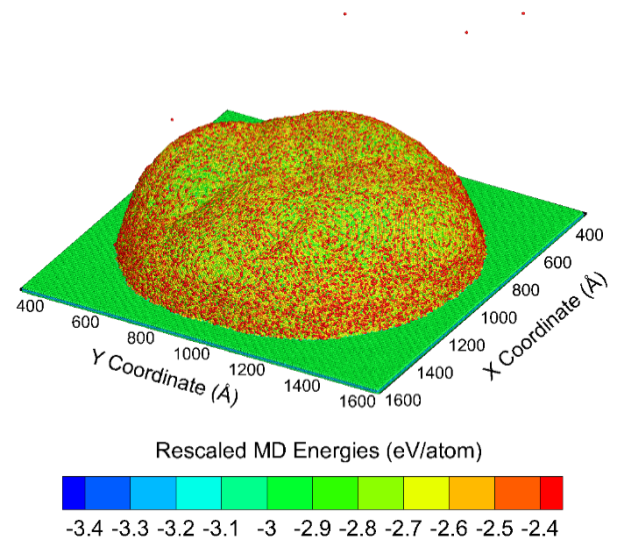
**

(a) MD (150 ps) (f) L2-QCGD (150 ps)

**Supplementary Figure S2: MD vs QCGD splat morphologies.** Comparison of the final splat morphologies between (a) MD (left) and (b) L2-QCGD (right) at a simulation time of t=150 ps. The atoms are colored on basis of their individual total energies for each atom/R-atoms.


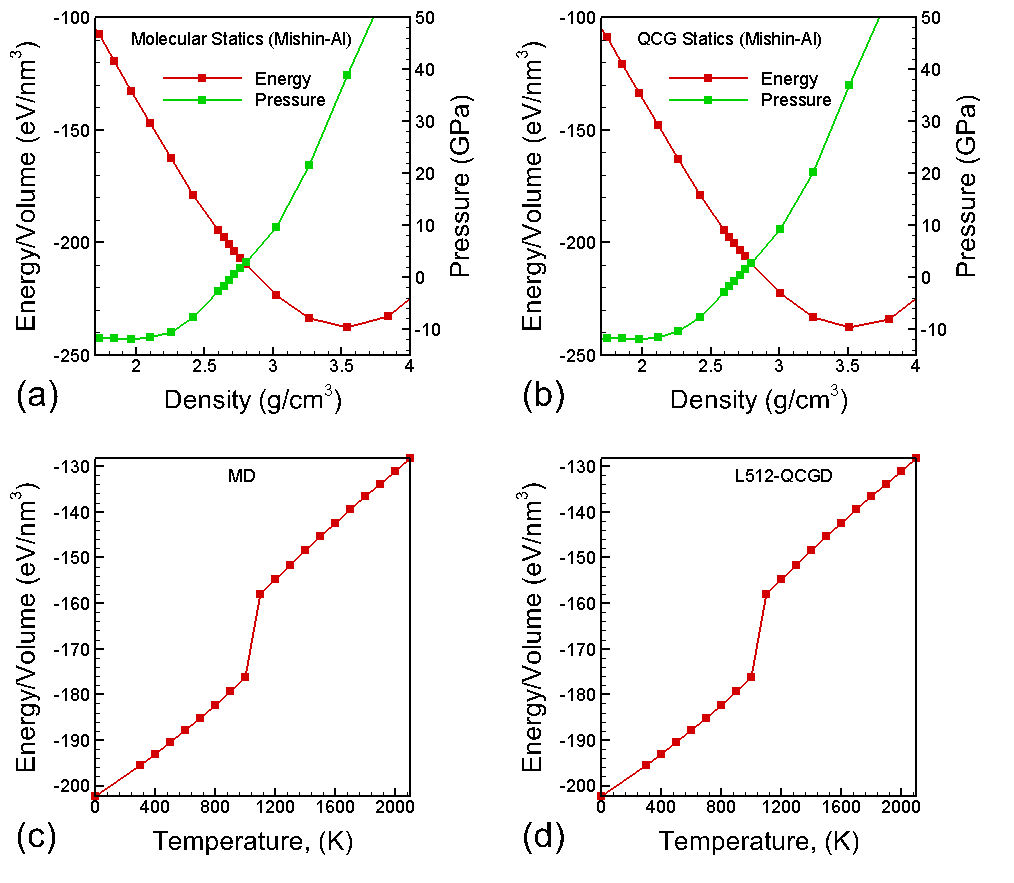


**Supplementary Figure S3: QCGD scaling relationships.** The equation of state (EOS) curve showing the variation of energy per unit volume and pressure with density of FCC Al calculated using (a) original and (b) L512-QCGD scaling relationships for the EAM potential. The comparison of the thermal dependence of the energy per unit volume using MD and L512-QCGD simulations is shown in (c) and (d), respectively

**
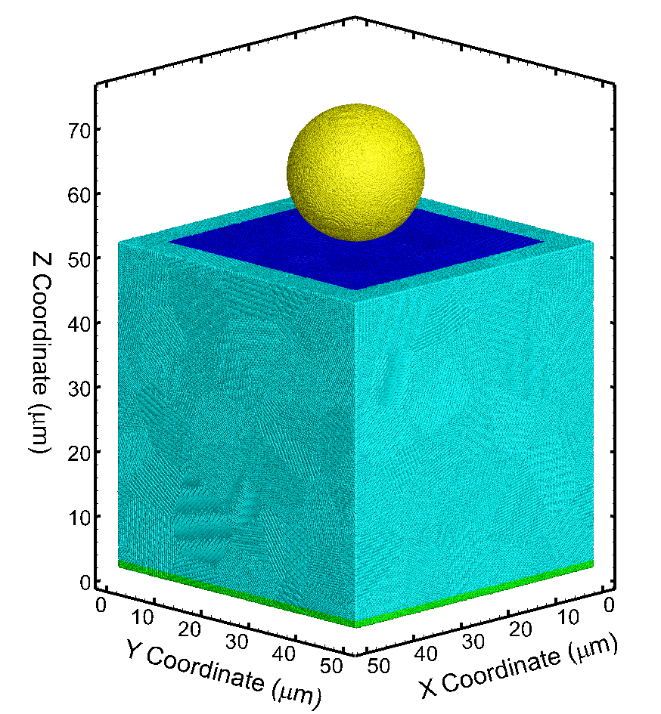
**

**Supplementary Figure S4: Setup for cold spray impact.** The initial setup for the QCGD-L512 single particle impact of a 20 µm polycrystalline aluminum sphere (yellow) over a 50 µm x 50 µm x 50 µm substrate (blue/light blue). A damping boundary region (light blue) is used to absorb the shock wave generated due to impact and a rigid boundary region (green) is used at the bottom of the substrate to prevent displacement of substrate due to impact.


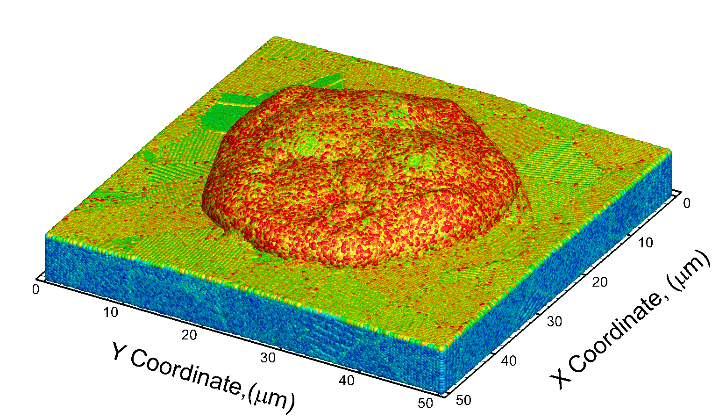

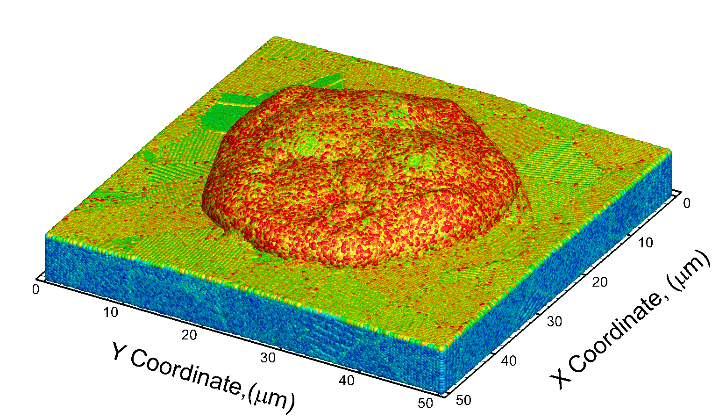


**8 μm**

**5 μm**

**5 μm**

(a) (b)

**Supplementary Figure S5: Temperature and Pressure Analysis**. Representative images showing of the cross-sectional portion of the whole system used for analyses of (a) pressure evolution, and (b) temperature evolution.


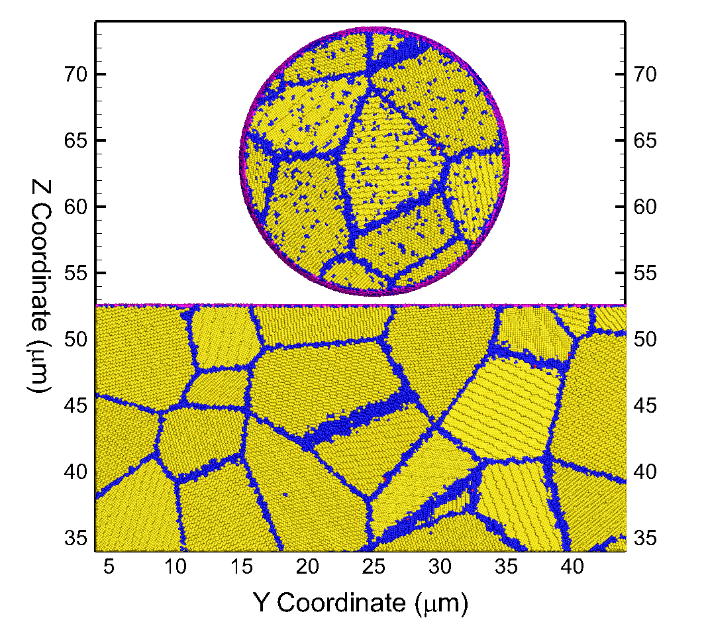

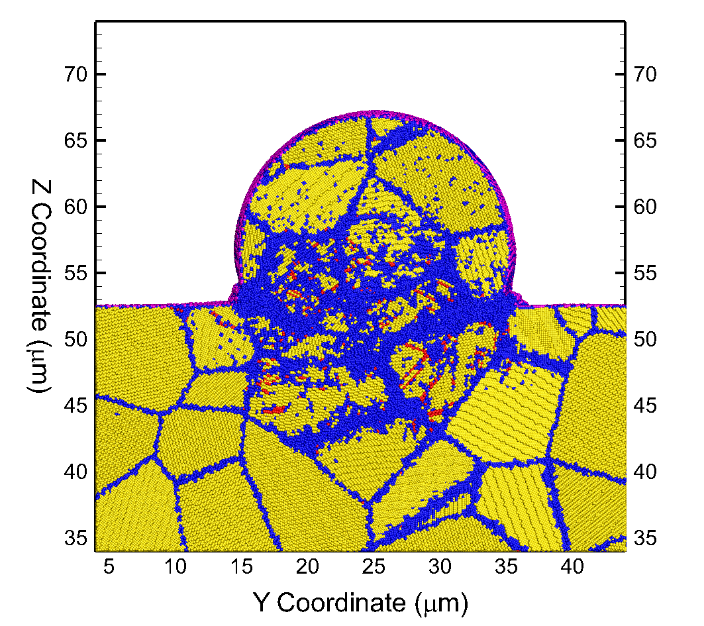


(a) (b)


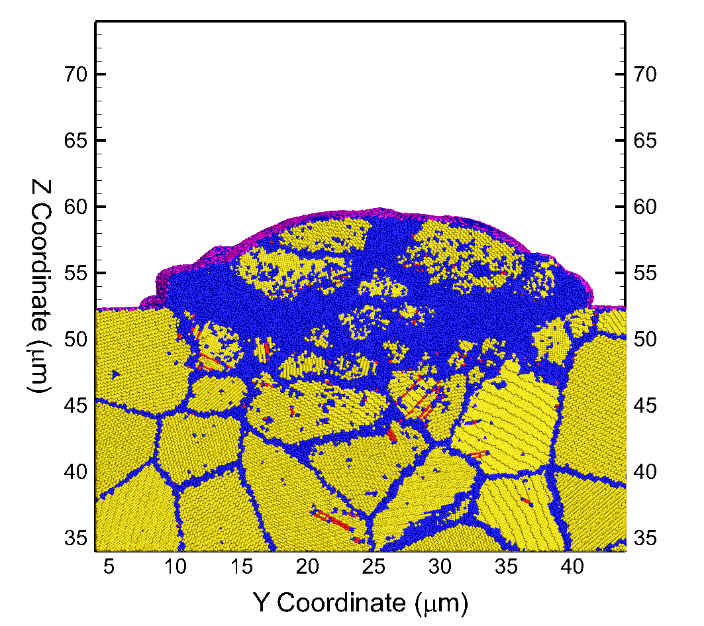

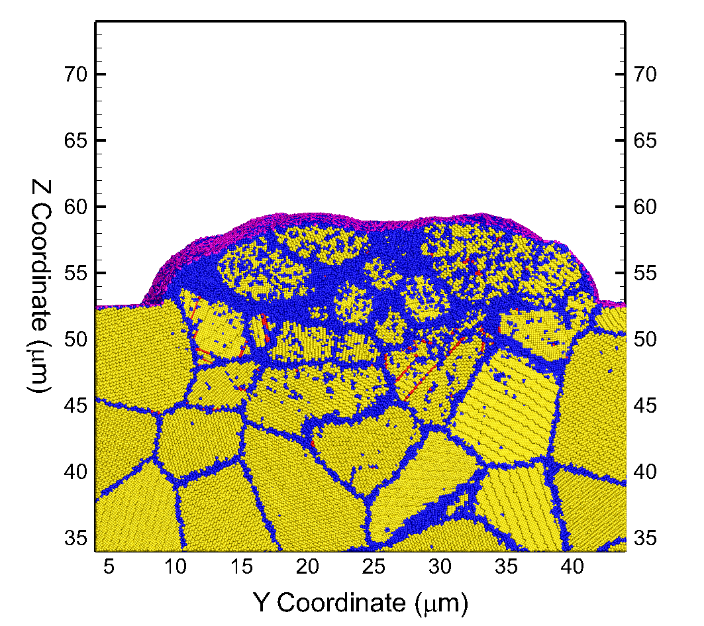


(c) (d)

**Supplementary Figure S6: Microstructural Evolution.** Defect analyses plots showing a thin section around the center of the system for the case of *v_i_*=1600 m/s for times, (a) t = 0 ns, (b) t = 4 ns (incipient “jetting”), (c) t = 12 ns and (d) t = 40 ns. The atoms are colored here based on Common neighbor analysis and yellow atoms represent fcc stacking, red atoms represent hcp stacking, purple atoms represent a surface and blue atoms represent a disordered structure.

*
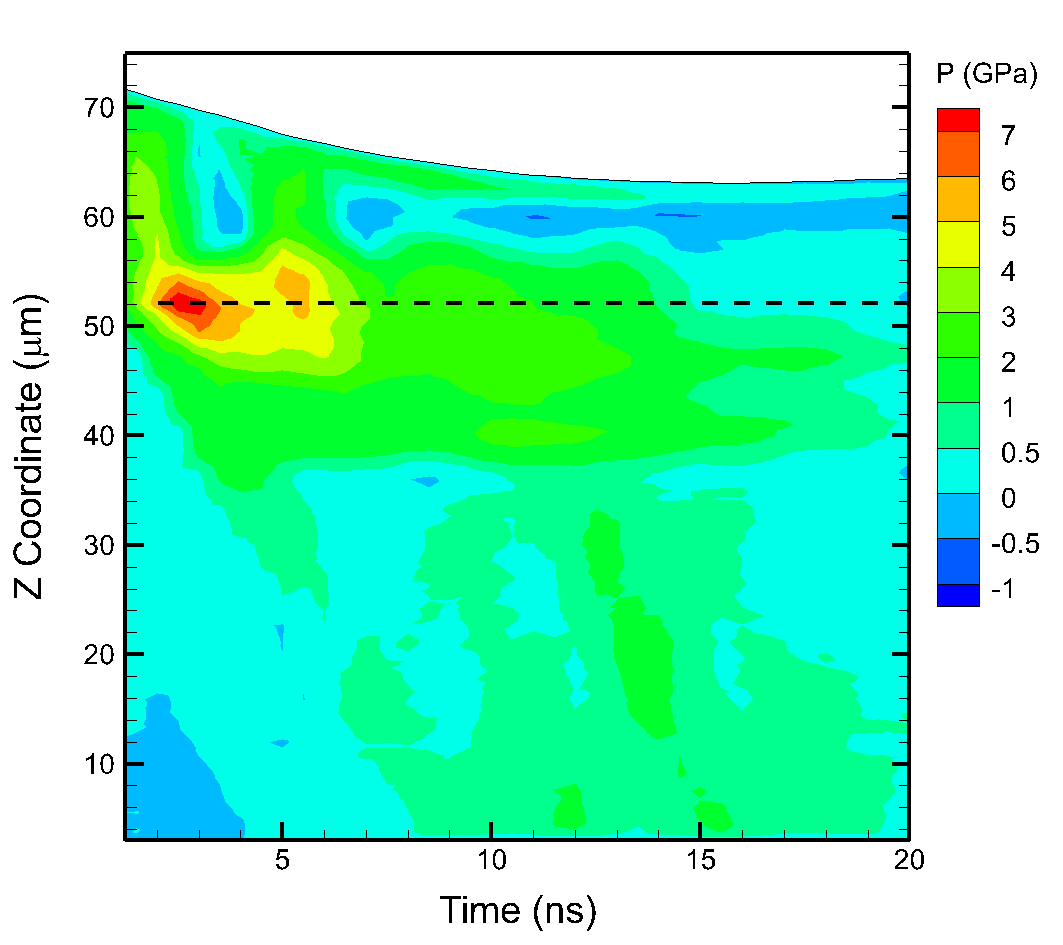
* *
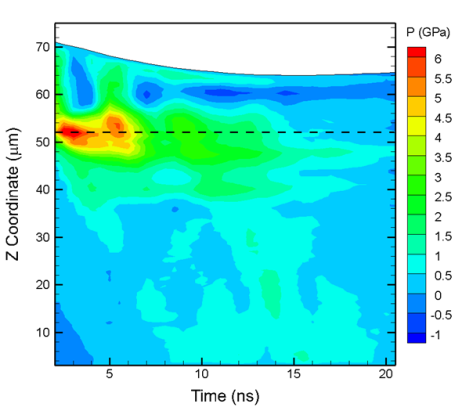
* *
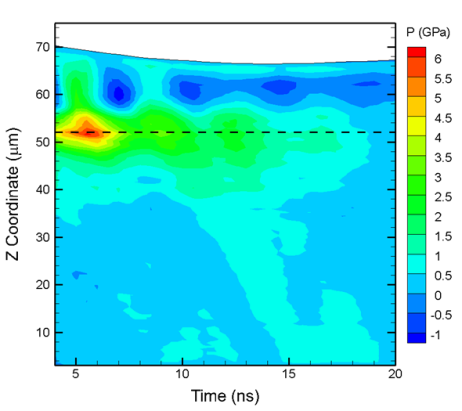
*

(a) 700 m/s (b) 1000 m/s (c) 1100 m/s

*
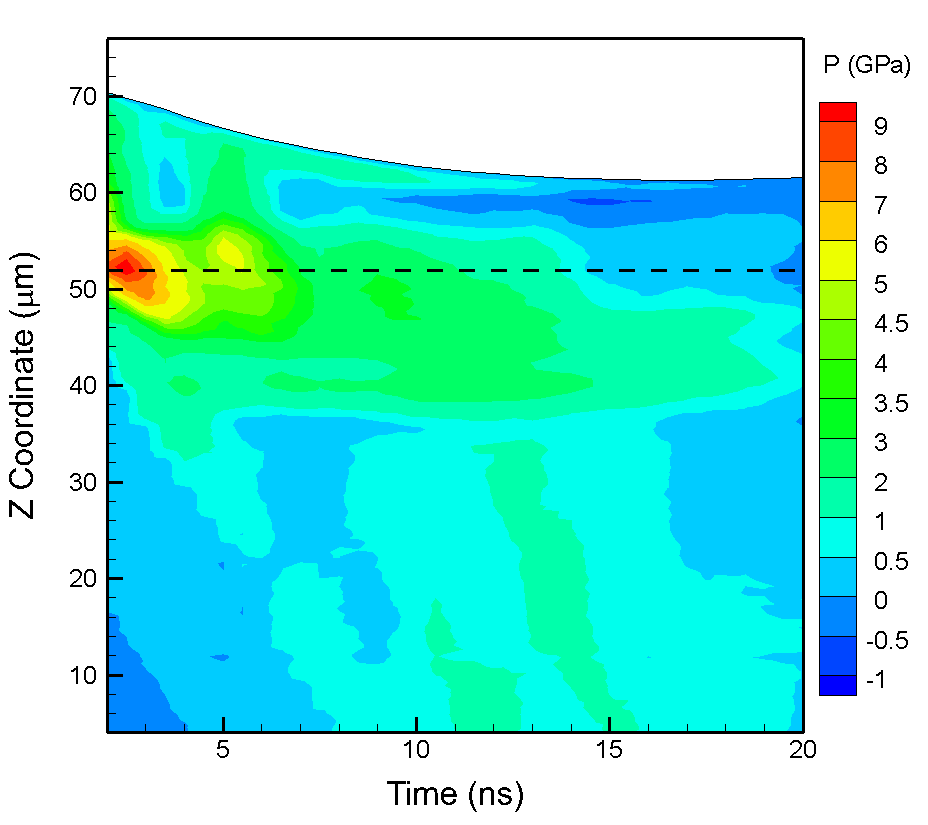
* *
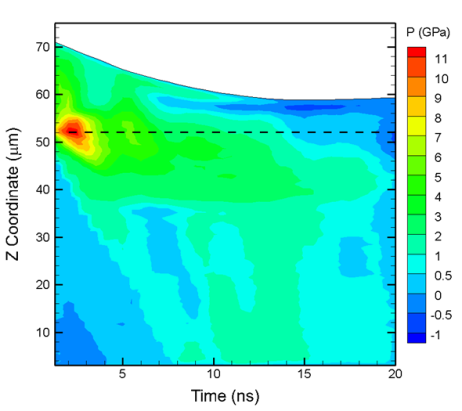
* *
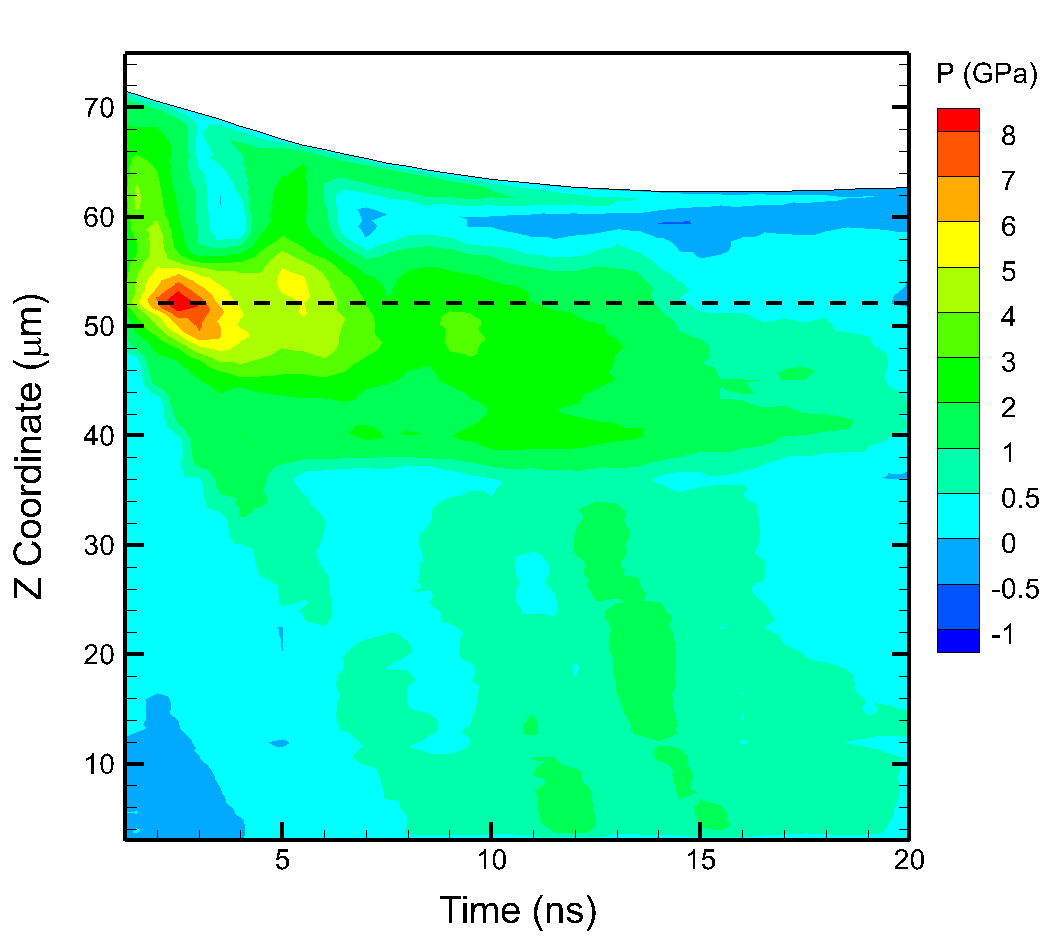
*

(d) 1200 m/s (e) 1300 m/s (f) 1600 m/s

**Supplementary Figure S7: Pressure dissipation at various impact velocities.** Evolution of pressures at the center of the particle and across the depth of the substrate for impact velocities of (a) 700 m/s, (b) 1000 m/s, (c) 1100 m/s, (d) 1200 m/s, (e) 1300 m/s and (f) 1600 m/s (dashed line denotes the maximum height of the substrate)

**Supplementary References**

1. [] Y. Mishin, D. Farkas, M.J. Mehl, and D.A. Papaconstantopoulos, Interatomic potentials for monoatomic metals from experimental data and ab initio calculations, *Phys. Rev. B* **59**, 3393 (1999). [↑](#endnote-ref-1)
2. [] A. M. Dongare, Quasi-coarse-grained dynamics: modelling of metallic materials at mesoscales, *Phil. Mag.* **94**, 3877-3897 (2014). [↑](#endnote-ref-2)
3. [] G. Agarwal, R. R. Valisetty, R. Namburu, A. M. Rajendran and A. M. Dongare, “The Quasi-Coarse-Grained Dynamics Method to Unravel the Mesoscale Evolution of Defects/Damage during Shock Loading and Spall Failure of Polycrystalline Al Microstructures”, *Sci. Rep.* **7**, 12376 (2017). [↑](#endnote-ref-3)
